# Comparison of Standard Culture-Based Method to Culture-Independent Method for Evaluation of Hygiene Effects on the Hand Microbiome

**DOI:** 10.1128/mBio.00093-17

**Published:** 2017-03-28

**Authors:** C. Zapka, J. Leff, J. Henley, J. Tittl, E. De Nardo, M. Butler, R. Griggs, N. Fierer, S. Edmonds-Wilson

**Affiliations:** a GOJO Industries, Inc., Akron, Ohio, USA; b Department of Ecology & Evolutionary Biology, Cooperative Institute for Research in Environmental Sciences, University of Colorado, Boulder, Colorado, USA; c BioScience Laboratories, Inc., Bozeman, Montana, USA; Icahn School of Medicine at Mount Sinai

**Keywords:** hand, hygiene, microbiome methods, skin bacteria, skin microbiome

## Abstract

Hands play a critical role in the transmission of microbiota on one’s own body, between individuals, and on environmental surfaces. Effectively measuring the composition of the hand microbiome is important to hand hygiene science, which has implications for human health. Hand hygiene products are evaluated using standard culture-based methods, but standard test methods for culture-independent microbiome characterization are lacking. We sampled the hands of 50 participants using swab-based and glove-based methods prior to and following four hand hygiene treatments (using a nonantimicrobial hand wash, alcohol-based hand sanitizer [ABHS], a 70% ethanol solution, or tap water). We compared results among culture plate counts, 16S rRNA gene sequencing of DNA extracted directly from hands, and sequencing of DNA extracted from culture plates. Glove-based sampling yielded higher numbers of unique operational taxonomic units (OTUs) but had less diversity in bacterial community composition than swab-based sampling. We detected treatment-induced changes in diversity only by using swab-based samples (*P* < 0.001); we were unable to detect changes with glove-based samples. Bacterial cell counts significantly decreased with use of the ABHS (*P* < 0.05) and ethanol control (*P* < 0.05). Skin hydration at baseline correlated with bacterial abundances, bacterial community composition, pH, and redness across subjects. The importance of the method choice was substantial. These findings are important to ensure improvement of hand hygiene industry methods and for future hand microbiome studies. On the basis of our results and previously published studies, we propose recommendations for best practices in hand microbiome research.

## IMPORTANCE

The hand microbiome is a critical area of research for diverse fields, such as public health and forensics. The suitability of culture-independent methods for assessing effects of hygiene products on microbiota has not been demonstrated. This is the first controlled laboratory clinical hand study to have compared traditional hand hygiene test methods with newer culture-independent characterization methods typically used by skin microbiologists. This study resulted in recommendations for hand hygiene product testing, development of methods, and future hand skin microbiome research. It also demonstrated the importance of inclusion of skin physiological metadata in skin microbiome research, which is atypical for skin microbiome studies.

## INTRODUCTION

Hands represent a critical target for microbiome studies because they have a unique role in transferring microbes, including beneficial microbes and pathogens, on one’s own body, between individuals, and between individuals and touched surfaces (1). With growing recognition of the essential role of nonpathogenic bacteria in human health, scientists have increasingly focused on the nonpathogenic, potentially beneficial microbes that live on the skin and hands (2, 3). Scientists are also exploring the potential for hand microbiome analyses to enable personal identification using microbial signatures left behind when surfaces are touched (4, 5). Therefore, it is likely that studies focused on the hand microbiome will continue to increase in number and importance, driving the need for more standardized methods.

The use of culture-independent methods to characterize microbial communities has increased in recent years due to their practical benefits. The primary advantage of culture-independent methods is their ability to readily identify a large proportion of the bacterial diversity that can be difficult to observe with culture-based studies. The currently used culture-based methods, although limited in breadth with respect to the types of organisms detected, have the advantage of quantifying absolute cell abundances of the culturable living microbes. The increase in use of culture-independent methods, namely those based on DNA sequencing, has been driven by advances in sequencing technologies and bioinformatic analysis tools and a reduction in the cost of conducting such studies (6, 7). In particular, the use of 16S rRNA gene sequencing methods has become routine in microbiology studies. For these reasons, culture-independent methods are expected to continue to be used more frequently in many fields, including hand hygiene effect research and product development. Since these methods are relatively new in comparison with culture-based microbiology assessments, they are still evolving and not yet standardized (6–8).

Hand hygiene is one of the most important tools available for reducing the spread of pathogens in health care and community settings (9–13). In the hand hygiene industry, efficacy assessments of hand hygiene products (e.g., antiseptic hand wash, antiseptic hand rub [sanitizer], and surgical hand preparation) have well-established standard protocols developed by ASTM International and the Committee for European Norms. The U.S. Food and Drug Administration (14) requires the use of these ASTM standards. The ASTM *in vivo* methodologies rely solely on measurement by culture-based techniques and fall into two categories. The techniques in one category are designed to evaluate effectiveness of hand hygiene products to remove transient pathogens from hands (e.g., ASTM E1174 [15], ASTM E2755 [16], and ASTM E2946 [17]). The subjects’ hands are experimentally contaminated with the test organism (a surrogate marker of a pathogen) before the test formulation is applied. In the techniques in the second category, which applies to surgical scrubs (e.g., ASTM E1115 [18]), the objective is to evaluate the hand hygiene product for its ability to reduce resident bacteria on hands. In both categories, hands are sampled using the “glove-based” methodology by vigorous massage in loose-fitting gloves with an eluent for 1 min, after which the eluents are assayed for bacteria using culture-based methods (10).

The (glove-based) sampling method employed traditionally in the hand hygiene industry differs from those typically used in skin microbiome characterization studies (swab-based sampling). For example, hands are washed with a nonantimicrobial soap prior to testing to remove excess transient bacteria in the ASTM standard hygiene methods, but this practice is uncommon in skin microbiome studies. Standardized hand hygiene methods prohibit participants from exposing their hands to any antimicrobials for days prior to sampling to reduce the confounding effects from other products, whereas this practice is uncommon in skin microbiome studies. To date, most skin microbiome studies have been ecological surveys, not controlled laboratory studies, designed to assess the effects of a specific perturbation, such as hand hygiene. Another fundamental difference in study design is sample timing. With swabs, it is feasible to sample skin immediately before and immediately after an intentional perturbation because swab-based sampling is not expected to drastically change the skin microbiota or physiological condition. However, glove-based hand sampling presumably changes the bacterial population and skin condition due to the vigorous nature of this approach. Thus, when glove-based sampling is used to sample resident bacteria on hands, such as in the ASTM E1115 surgical scrub method, the “baseline hygiene” hand sampling must be done days prior to the “immediately after” sampling to allow for the resident bacteria and skin to return to baseline conditions before the hand can be resampled. Therefore, there are a number of important variables to consider when designing a hand sampling study.

Methodological choices are known to affect observed results in human microbiome studies (7). Such factors include subject inclusion and exclusion criteria, how skin is sampled, genetic material extraction approach, storage solutions and conditions, sequencing platform, regions sequenced, and bioinformatics data processing and analysis, among others (7, 19, 20). A study performed by Rosenthal et al. (21) was the first microbiome study to use the glove-based hand sampling method and to result in the observation that glove-based samples yielded data that were more similar at different time points for the same individuals than the data from swab-based samples, which had greater variation. In another skin sampling method comparison study, samples taken by scrape, swab, and biopsy produced similar results (22). Sequencing methodologies based on amplification of the V1-V3 hypervariable region of the 16S rRNA gene also have been shown to report community composition results that are different from the results from other regions (20).

The development of robust standard culture-independent methods for characterization of hand microbiota to be used for the assessment of hand hygiene products is needed. More robust studies must characterize differences between current standardized hand hygiene protocols and the emerging culture-independent skin microbiota characterization method options. Our report compares results obtained from standard culture-based methods with those obtained from culture-independent skin microbiome characterization methods for assessment of the impact of hygiene products on the hand microbiome. In addition, we assessed the relationships among hand microbiota and physiological skin characteristics. Specifically, we characterized the hands of 50 individuals before treatment and immediately, 24 h, and 7 days after treatment with a hygiene intervention. We sampled hands using both swab-based and glove-based methods and characterized bacteria using culture plating (aerobic and anaerobic), culture-independent 16S rRNA gene sequencing, and sequencing of the organisms grown on the culture plates. We provide a framework outlining many of the methodological decisions to be considered in the design, execution, and data analyses of a hand microbiome study.

## RESULTS

### Characterization and comparison of the skin and microbiota of hands at baseline.

**FIG 1:**
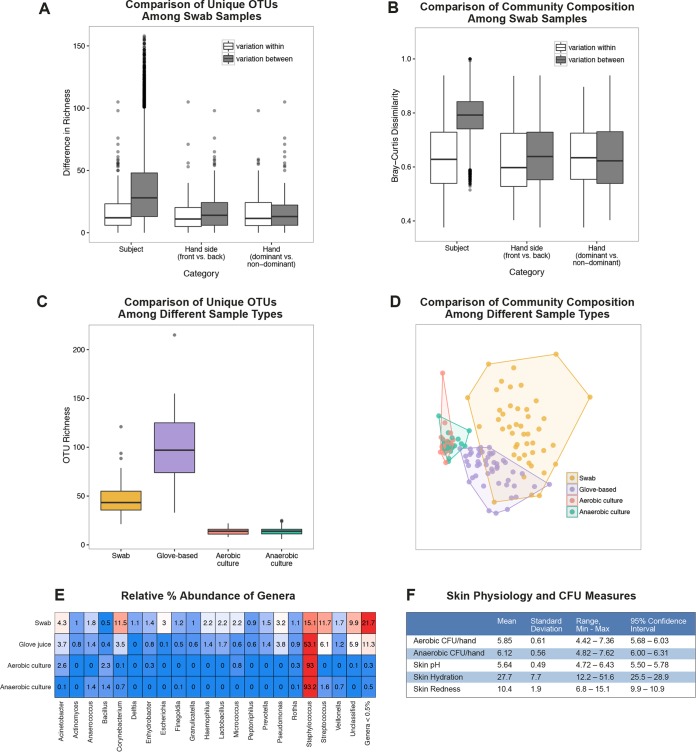
Characterization of the skin and microbiota of hands at baseline. (A and B) OTU richness (A) and Bray-Curtis dissimilarity (B) in bacterial community composition in samples from swabs collected immediately before hygiene treatment between samples across subjects versus within subjects, between samples from different sides of hands versus between samples from the same side of hands within subjects, and between samples from different hands versus between samples from the same hand within subjects. Boxplots represent minimum values, first quartiles, medians, third quartiles, maximum values, and outliers. (C and D) Diversity measured as richness of bacterial OTUs (C) and diversity (D) illustrated using nonmetric multidimensional scaling (NMDS) ordination showing differences in bacterial community structure observed from swabs, glove-based samples, and anaerobic culture plating of glove-based samples and aerobic culture plating of glove-based samples. (E) Relative percent abundance of genera recovered at baseline by swab, glove-based, aerobic plating, and anaerobic plating. Blue represents lower relative abundances, and red represents higher relative abundances. (F) Baseline results for aerobic and anaerobic bacterial CFU levels and skin pH, hydration, and redness measures. Min, minimum; Max, maximum. *n =* 50 for all measures.

We first compared numbers of unique operational taxonomic units (OTUs) and community compositions (based on Bray-Curtis dissimilarities) for the hands of a given subject and between subjects’ hands. We compared the levels of variability in diversity within and between subjects, between hand sides (palmar versus dorsal), and between hand dominances among the swab-based samples (Fig. 1A and B). Variation between subjects was greater than for within-subject samples and significant for numbers of unique OTUs (analysis of variance [ANOVA]; *P* < 0.001). The relative differences in numbers of unique OTUs between hand samples taken from different hands of the same subject or different sides of the same hand of a subject were not statistically significant (ANOVA; *P* = 0.60 [hand side], *P* = 0.86 [for dominance]). We also observed differences in bacterial community composition across subjects, as within-subject variation was significantly lower than variation between individuals (permutational multivariate analysis of variance [PERMANOVA], *P* = 0.001; *r*^2^ = 0.50). We observed no effect of hand dominance (PERMANOVA; *P* = 0.36). Hand side (palmar versus dorsal) had a significant (PERMANOVA; *P* = 0.002) but weak (*r*^2^ = 0.006) correlation with bacterial composition.

Next, we compared the differences in diversity among glove-based, swab-based, and aerobic and anaerobic culture growth plate wash sequencing samples. Sample collection and recovery method had a significant impact on the observed numbers of unique OTUs (Kruskal-Wallis test; *P* < 0.001) and on bacterial community composition (PERMANOVA; *P* < 0.001; *r*^2^ = 0.38) (Fig. 1C and D). As expected, sequencing of plate washes of the cultures grown from either aerobically or anaerobically grown bacteria showed significantly lower numbers of unique OTUs and diversity in bacterial community composition, indicating that plating underestimates the diversity of the hand skin bacterial community. Glove-based samples had the greatest richness, resulting in recovery of, on average, 97.6 unique OTUs per hand per 1,000 sequences. Culture plate washes had the lowest richness, with approximately 14.6 OTUs per hand per 1,000 sequences, whereas the richness for swab-based samples averaged 48.3 OTUs per hand per 1,000 sequences. Swab-based samples, however, had a greater range in bacterial composition among participants than glove-based samples. *Staphylococcus* was the most abundant genus, comprising 93% of the recovered sequences from plate washing and 53% of the recovered sequences from glove-based samples, on average (Fig. 1E). Other genera most commonly observed were *Streptococcus*, *Corynebacterium*, *Pseudomonas*, and *Acinetobacter*. Swab-based samples contained the highest proportion of rare OTUs, with 21.7% of sequences represented by OTUs that were each present at <0.5% relative abundance, compared with 11.3% rare OTUs in glove-based samples and <1% in plate washes. OTUs unclassified at the genus level comprised 9.9% of the swab-based samples, whereas they comprised 5.9% of the glove-based samples.

We also characterized the levels of culturable bacteria of the participants’ hands at baseline (Fig. 1F). The level of viable bacteria recovered at baseline on aerobic plates was, on average, 5.85 log_10_ CFU per hand, with a range from 4.42 to 7.36 log_10_ CFU. On anaerobic plates, the level of viable bacteria recovered at baseline was, on average, 6.12 log_10_ CFU per hand, with a range of 4.82 to 7.62 log_10_ CFU.

Because bacterial colonization is driven by the ecology of the skin surface, we measured baseline skin metadata (pH, hydration, and redness) to determine whether these variables correlate with bacterial community composition. Skin hand baseline pH measurements ranged from 4.72 to 6.43, averaging 5.64; hydration ranged from 12.2 to 51.6, averaging 27.7; and redness ranged from 6.8 to 15.1, averaging 10.4. All of these measurements were as expected for the healthy participant population.

### Impact of methodology on observed hygiene effects.

**FIG 2:**
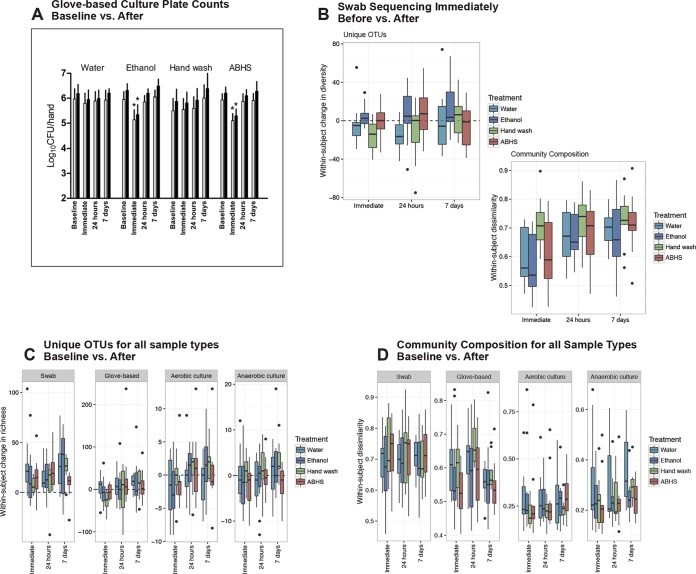
Impact of methodology on observed hygiene effects. (A) Quantity of viable aerobic (light bars) and anaerobic (dark bars) bacteria recovered per hand at baseline by glove-based sampling immediately after, 24 h after, and 7 days after use of an ABHS, hand wash, ethanol control, or water control. Asterisks indicate significant differences observed compared with baseline (*P* < 0.05, ANOVA). Bars indicate means and error bars the 95% confidence intervals. (B) The OTU richness and Bray-Curtis dissimilarity within-subject diversity in community composition between samples collected immediately before and samples collected immediately, 24 h, and 7 days after use of an ABHS, hand wash, ethanol control, or water control as assessed with swab samples. (C and D) Within-subject OTU counts (C) and dissimilarity in community composition (D) observed from swabs, glove-based samples, and anaerobic and aerobic culture plating of glove-based changes between samples collected at baseline and samples collected immediately, 24 h, and 7 days after use of an ABHS, hand wash, ethanol control, or water control. *n* values for test groups are as follows: water = 14, ethanol = 13, hand wash = 11, and ABHS = 12.

We compared the observed effects of four different hand hygiene treatments on the hand microbiota among different microbiota sampling and measurement approaches. The observed impacts of hygiene intervention differed considerably by method (Fig. 2). First, we assessed the effect of hygiene on levels of culturable bacteria (Fig. 2A). The nonantimicrobial hand wash and water rinse controls did not significantly impact viable aerobic or anaerobic bacterial counts at any time point. In contrast, the alcohol-based hand sanitizer (ABHS) and ethanol control performed similarly; both significantly reduced the levels of viable aerobic bacteria immediately after product use (by 0.84 log_10_ CFU reductions and 0.81 log_10_ CFU reductions from baseline, respectively) (ANOVA; *P* < 0.05). Between 5.10 log_10_ CFU and 5.36 log_10_ CFU viable resident aerobic bacteria remained on hands immediately after use of the ABHS and ethanol. Similarly, the ABHS and ethanol control significantly reduced (ANOVA; *P* < 0.05) the levels of viable anaerobic bacteria immediately after product use by 0.90 log_10_ CFU reductions and 0.95 log_10_ CFU reductions from baseline, respectively. The reductions were temporary for both aerobic and anaerobic bacteria, as the levels of culturable bacteria were not significantly different between baseline and samples taken 24 h or 7 days post-hygiene treatment.

Next, the effect of hand hygiene on bacterial community compositions was investigated. Swab-based samples that were obtained immediately before treatment were compared with those collected at multiple time points following treatment (Fig. 2B). Paired *t* tests of within-subject OTU richness, before versus after treatment, for each treatment and time point swabbed showed that the hand wash treatment caused a significant decline in richness immediately after treatment (*P* = 0.01). Neither the ABHS nor either control (water or ethanol) treatment resulted in a significant change immediately after treatment. The OTU richness observed from swab-based samples 24 h following water treatment was significantly decreased also (*P* = 0.003). Within-subject dissimilarity in community composition between pretreatment and immediately posttreatment swab-based samples was significantly greater among subjects receiving the nonantimicrobial hand wash than among those receiving an ABHS or either control treatment (ANOVA; *P* < 0.001). This difference was not significant after 24 h or 7 days following treatment.

Finally, we studied the effect of hygiene on the bacterial communities obtained using glove-based sampling. Within-subject numbers of unique OTUs and bacterial community compositions observed from swab-based samples, glove-based samples, and anaerobic and aerobic culture plating of glove-based sample changes between samples collected at baseline, as well as samples collected immediately, 24 h, and 7 days after use of an ABHS, nonantimicrobial hand wash, ethanol control, and water control, are illustrated in Fig. 2C and D. In this analysis, there were two different “before” and “after” hygiene treatment comparisons made. The first comparison was modeled after the standard hand hygiene method in which the “baseline” measures were taken days prior to the hygiene treatment. This first analysis of data for the baseline versus after treatment was performed for all four sample types collected: swab-based samples, glove-based samples, anaerobic plate culture washes, and aerobic plate culture washes. In the second comparison, the “before” measures were taken immediately before, and on the same day as, the hygiene treatment. Since only the swab-based samples could be taken immediately before the hygiene treatment, the second analysis was performed only for swab-based samples. No significant changes were observed in bacterial community composition as a result of hygiene use for any of the sample types in comparisons of baseline samples (PERMANOVA; *P* > 0.05). Interestingly, there was a systematic trend of an increase in numbers of unique OTUs across most time points in swab-based samples but not in glove-based samples or culture plate washes. The richness measured at baseline for swab-based samples was generally lower than the richness in all swab-based samples taken at all of the “after” time points. The other sample types (glove-based samples and plate washes) did not show the same increase in richness as the swab-based samples post-hygiene treatment.

### Associations among measures of microbiota, skin physiology, and hygiene effects.

**FIG 3:**
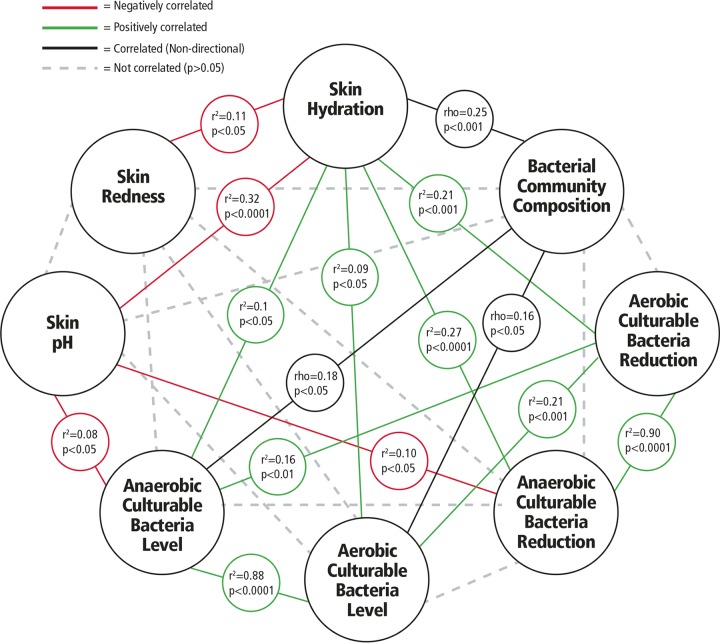
Associations among measures of microbiota, skin physiology, and hygiene effects. Results of statistical comparisons among eight measures are illustrated. Relationships between skin metrics and bacterial community composition were assessed by matching the swab sample collected from the back of the same hand in which the skin metrics were observed and using Mantel tests with Spearman correlation. Bacterial community composition and culturable bacterial level results from glove-based samples were correlated using Mantel tests with Spearman correlation. Correlations among the skin measures, plate counts, and log_10_ reductions for the time point immediately after hygiene treatment were identified using linear regression, which reports significance as well as the direction of correlation (positive or negative). Bacterial community composition is a categorical measure, not a numerical metric, so only data for the significance (not direction) of correlations are reported. *n* = 50 for all measures.

We examined whether there were associations within the broad set of measures taken using baseline hand samples (metadata). Two different correlation measures were needed for this analysis. Bacterial community composition was compared with all other measures using Mantel tests with Spearman correlations. All of the other measures were assessed by linear regression, which reports correlations as *r*^2^ values. Figure 3 shows the correlations observed within the metadata, including skin physiological state (pH, hydration, and redness), microbiota composition (bacterial community composition and levels of culturable anaerobic and aerobic bacteria), and observed hygiene effects (reductions in levels of anaerobic and aerobic culturable bacteria immediately after treatment).

The strongest correlations were between the baseline levels of anaerobic and aerobic culturable bacteria (*r*^2^ = 0.88; *P* < 0.0001) and between observed reductions in levels of culturable bacteria immediately after any hygiene treatment versus baseline (*r*^2^ = 0.90; *P* < 0.0001) as illustrated in Fig. 3. This was not surprising as baseline characterization performed with culture plating demonstrated that the types of bacteria recovered aerobically and anaerobically were similar. The numbers of bacteria recovered under anaerobic conditions were significantly greater than those recovered under aerobic conditions (paired two-tailed *t* test, *P* < 0.0001).

Hydration was the only variable that was associated with all other microbiota and skin physiological measures. Baseline skin hydration correlated with redness, pH, bacterial community composition, levels of culturable bacteria (anaerobic and aerobic), and the observed immediate effects of hand hygiene on levels of bacteria (anaerobic and aerobic). There was a significant (*P* < 0.05) but weak (Mantel rho = 0.16 for aerobic bacteria and 0.18 for anaerobic bacteria) relationship between differences in bacterial load (CFU) and community composition at baseline. In addition to hydration, skin pH correlated significantly (*P* < 0.05), but weakly, with levels of anaerobic bacteria (*r*^2^ = 0.08) and reductions of levels of anaerobic bacteria immediately after hygiene treatment (*r*^2^ = 0.10) but not with levels of aerobic culturable bacteria.

## DISCUSSION

The effects of hand hygiene practices on the skin and hand microbiome are an important area of study, but relatively few robust studies have been conducted to fully elucidate the optimal protocols to ensure repeatability, precision, and accuracy. Well-established protocols are currently available from the hand hygiene industry to assess the effect of hand antiseptics on the culturable component of hand bacteria (14). However, the objective of these methods is to evaluate the reduction of only the culturable bacterial species on hands, not to assess the impact on the entire bacterial community. Thus, culture-independent methodologies have been increasingly used for the analysis of the hand microbiome.

We compared methods used in the hand hygiene industry to those used in skin microbiome studies for the assessment of the impact of hygiene interventions on the hand microbiota. Through the comparison of methods, we identified important insights that strongly suggest that combining elements of both approaches is a preferred method for assessing the hand resident bacterial community and the effects of hygiene practices on the hand microbiome.

### Interpretation of key findings.

Glove-based sampling recovered more unique OTUs from individual hands than swab-based sampling. Furthermore, the bacterial community compositions recovered in glove-based samples were more similar among participants, whereas swab-based samples demonstrated greater variability in bacterial community compositions among participants (as shown in Fig. 1D). Glove-based sampling results at baseline had higher richness than swab-based results, which indicates there were greater numbers of unique OTUs. We hypothesize that this pattern is the result of glove-based sampling recovering greater total biomass and bacterial communities, as the total surface area of the hand that is sampled by glove-based sampling is larger than the area involved when swabs are used. Glove-based sampling represents more of the entire hand, including the interdigital spaces and fingernails, than swab-based sampling does. The variations in bacterial community compositions observed among the 50 participants were much greater for the swab-based samples than for the glove-based samples, perhaps indicating that the glove-based method recovers resident populations that are more similar between people than previously thought on the basis of results of swab-only sampling studies (23, 24). A potential criticism is that the swabbing was done on the hands prior to glove-based sampling, which may have influenced the study results. However, all hands were prewashed to remove transient bacterial populations prior to sampling, which would reduce the impact of this potential effect. Future studies conducted to directly compare glove-based sampling results with versus without prior swab-based sampling would be valuable; however, this potential limitation is outweighed by the benefit that this study design choice provided—enabling direct comparison of the same hand at the same time for glove-based sampling and swab sampling. Only swab-based samples (and not glove-based samples or culture plate washes) showed an increase in the number of different types of bacteria recovered after all hand hygiene treatments at each time point. These results cannot be conclusively explained. Possible reasons include hygiene treatment reducing the dominant OTU (thus increasing the apparent number of different OTUs), technician sampling technique variability, and/or variability in environmental conditions.

We observed interpersonal variability to be greater than intrapersonal variability, similarly to other studies (24–26). The types of bacteria recovered were similar to those seen in previous studies as reviewed by Edmonds-Wilson et al. (1), with the notable exception of a low (<0.5%) relative abundance of *Propionibacterium* species, which we attributed to primer bias and use of the V4 region (20). According to some researchers, use of the V1-V3 region of 16S may be preferred for skin studies when whole-genome or shotgun approaches are not feasible (20).

Previous studies have shown a significant difference in bacterial community composition between the dominant and nondominant hands of individuals (23, 27); however, our study did not support this finding. Previous studies did not include a prewash of the subjects’ hands to reduce transient bacteria; such a prewash presumably allows characterization of primarily resident bacteria. It is possible that the differences observed in previous studies were driven by the transient microbial population, which might be expected to be more abundant on the dominant hand, while this study indicated that the more stable resident populations are more similar among people.

Results showed that the microbiome compositions for swab samples from the palmar and dorsal sides of the hands were similar. This was unexpected because the skin structure and physiology are distinct for the palmar versus dorsal regions of the hand (28, 29). However, this difference may have been underestimated since subjects washed their hands before sampling, spreading bacteria from all areas of both of their hands prior to our sampling and potentially homogenizing the hand microbial communities. Alternatively, since our study included a prewash to reduce levels of transient microbes while other studies did not, the differences observed between the palmar and dorsal surfaces in other studies may have been influenced by the presence of greater populations of transient microbes from touched surfaces on the palmar surfaces versus the back of the hands.

We wanted to determine if there were any relationships among baseline skin physiology, microbial counts, bacterial community composition, and observed impact of hygiene treatments. We included skin physiology measures because microbial colonization is driven by the ecology of the skin surface, which is highly variable depending on topographical location, endogenous host factors, and exogenous environmental factors, including moisture and pH (30, 31). Healthier skin is more hydrated and generally has lower pH (32, 33). Our findings, illustrated in Fig. 3, confirmed that there were several significant correlations identified among (i) bacterial community composition assessed by sequencing, (ii) aerobic culturable bacterial CFU level, (iii) anaerobic culturable bacterial CFU level, (iv) aerobic culturable bacterial population reduction immediately after hygiene use, (v) anaerobic culturable bacterial population reduction immediately after hygiene use, (vi) skin pH, (vii) skin hydration, and (viii) skin redness. A few of the correlations found were strong and significant, though there were many other additional significant but weak correlations with relatively small *r*^2^ and rho values that also were identified. However, the overall pattern observed among the weaker correlations in the collective data may suggest that these are meaningful trends.

Skin hydration appears to be the most critical variable for explaining variation in bacterial community composition and abundances and in other skin characteristics. The overall pattern of correlations suggests that individuals with higher skin hydration levels have lower pH, less redness, larger amounts of culturable bacteria (both aerobic and anaerobic), and bacterial community compositions that differ from those of persons with low hydration. Also, those with higher levels of skin hydration demonstrate greater reductions in populations of culturable bacteria (aerobic and anaerobic) when exposed to hygiene treatment. Future studies should aim to elucidate what is the cause and what is the effect: does more-hydrated skin result in higher levels of microbes and a particular microbial population, or does the presence of certain microbial populations result in more-hydrated, healthier skin?

Acidity was not strongly correlated with baseline microbiota composition of the hands. This was an unexpected finding since past studies suggested that pH is a key driver of microbial populations and can both affect and inhibit growth of certain organisms, such as *Staphylococcus* and *Streptococcus* species (34–36). This may have been an artifact of our subject population (all young females with healthy skin), for which pH was in a relatively narrow healthy range (between 4.72 and 6.43; mean = 5.64). Alternatively, it may have been an artifact of the effect of prewashing hands with soap, which may have inadvertently impacted the pH and made all of the participants’ pH measures more similar than would have been expected if no wash had been used.

### Effect of hygiene interventions on the hand microbiome.

Our results show that the methods used to assess the hand microbiome had a substantial effect on the observed impact of hygiene treatments. By culture methods, the ABHS and ethanol control had greater impact than the nonantimicrobial hand wash or water rinse control on the numbers of viable bacteria. The nonantimicrobial hand wash, however, had the greatest impact in assessment using bacterial community composition. These results align with previous studies that indicated that nonantimicrobial hand wash use can modify the microbiome (23, 37). No change in community composition, via glove-based sampling, was observed for any of the hand hygiene treatments. In contrast, swab-based sampling detected a change in bacterial OTUs and community composition as a result of hygiene treatment with a hand wash. Sequencing of both swab-based and glove-based samples showed that the use of ABHS had no more impact on diversity, as measured by OTU richness, or on the hand bacterial community composition, than did rinsing with water. This may indicate that hand hygiene interventions act on only the outer surface of the skin and do not change the resident populations below the outermost surface of the skin (38). However, in all instances, any effects were not lasting, as no significant differences were observed at the follow-up sampling time point of 24 h. It is likely that the microbiota returned in less than 24 h, but future studies will need to be conducted with shorter sampling periods (e.g., minutes and hours) to address that issue.

Our results also suggest that the hand microbiome is resilient with respect to changes caused by use of all hand hygiene products but that the effects of nonantimicrobial hand wash and those of ABHS use on the microbiota are not the same. Hand washing with a nonantimicrobial soap impacted the bacterial community composition, while use of an ABHS did not. Use of the hand wash did not change the levels of viable bacteria on the skin, while use of the ABHS did. However, greater than 100,000 CFU of resident bacteria remained on the hands after ABHS use, showing that hand hygiene in general, and the use of ABHS in particular, does not sterilize the skin.

### Recommendations for hand microbiome methods.

Based on our results and previously published studies, we propose a set of recommendations for best practices in hand microbiome research. Several best practices for all microbiome studies have already been outlined by Sinha et al. (19) and Goodrich et al. (7). We agree with the recommendations provided in those key references and encourage hand microbiome science studies to follow them. The remainder of this section focuses on additional recommendations that are specific to hand microbiome studies.

For all studies, we (i) recommend that hand microbiome studies include both culture and culture-independent methods, since each by itself presents an incomplete picture. Additionally, anaerobic plating is recommended, as it recovered greater total levels of bacteria than aerobic plating, but the two methods qualitatively delivered similar community compositions. Culture plating overrepresented the relative prevalence of the *Staphylococcus* species and underrepresented the overall diversity of the microbial community detected using culture-independent characterization. Since culture-independent sequencing is not able to differentiate between viable and dead microbes, the effect of test articles (ABHS and ethanol control) that kill bacteria may be underestimated. Plating is good for quantification and viable counts but detects only a fraction of the diversity of the community. While sequencing is better at showing the entire community, it is not effective in distinguishing living from inactive or dead microbes. In the future, the need for culture plating could be eliminated, if sequencing methods improve to become more quantitative, such as by using a spiking method (39), and lead to resolution of the problem of the inability to distinguish live and/or active microbes from dead and/or dormant bacteria, such as with the propidium monoazide (PMA) method (40) or SYTOX-based methods (41).

Based on the higher variability in the swab-based sampling technique, the ability to obtain greater biomass from glove-based sampling, and the hypothesis that glove-based samples are a better representation of the resident hand microbiota, we (ii) recommend glove-based sampling for most hand microbiome studies. This recommendation concurs with the conclusions of Rosenthal and colleagues (21). Glove-based sampling is preferred when characterization of the resident population is desired, while swab-based sampling may be appropriate for some studies aimed at assessing only a portion of the hand surface area when transient bacterial populations are the focus of the study. The cup scrub sampling technique (42) could be explored in future studies, as it may be a more direct comparison to swabbing, as it can also be done on smaller areas of the skin.

For studies aimed at assessing the resident hand population, we (iii) recommend inclusion of a prewash with a nonantimicrobial hand wash to remove transients prior to taking any measurements. Results are more likely to represent mostly resident microbiota after a prewash, while samples taken without a prewash are more likely to represent a mixture of resident and surface transient bacteria. If the study’s objective is to profile the transients, then a prewash is not needed. However, in assessing the impact of an intentional perturbation, such as hand hygiene, the prewash is important to reduce the background noise and variability introduced by surface transients.

We (iv) recommend that one hand be used to measure the hand microbiota condition before hygiene intervention and that the other hand of the same participant be used to measure immediately after hygiene product use. A key limitation in the current ASTM method (E1115-11) for assessment of the effect of hand hygiene on the resident bacteria is the confounding factor of time and the environmental exposures that the hands encounter. This is because the baseline is measured days prior to the post-hygiene sample. In our study, we chose to use the same hand for all glove-based sampling, following this ASTM approach. This is because we assumed, on the basis of previous work (23), that the microbiotas of the two hands of each participant would be markedly different. The similarity between dominant and nondominant hands observed in glove-based samples in our study suggests that it is acceptable to assume that the two hands are similar. We believe that the error introduced by taking baseline measurements on the same hand on different days prior to post-hygiene measurements (as recommended by standard ASTM hand hygiene methods) would be greater than the error introduced by taking measures using different hands but on the same day (i.e., taking measures from one hand immediately before the hygiene intervention and from the other hand immediately after the hygiene intervention).

Since hydration appeared to be the key driving factor in our study, we (v) recommend that hydration be measured in all skin microbiome studies, at a minimum, and that other measures such as pH are included, when feasible. Other skin physiological measures, such as transepidermal water loss measurements to assess skin barrier function, desquamation index analysis to measure skin cell shedding, and expert visual grading to assess overall skin condition, should also be considered for inclusion in future hand microbiome studies to further our understanding of the relationship between hand microbiota and skin health.

By comparing hand hygiene industry standard methods to protocols used by skin microbiome survey studies, our results revealed that researchers should use caution in applying only culture-independent methods to assess the effects of hygiene products. It is important to recognize that the suitability of emerging skin culture-independent microbiome methods to assess hygiene impacts has not yet been demonstrated. A better approach is likely to combine elements of both the existing culture-based standard methods with the culture-independent methods to assess microbial loads and microbial community composition in order to more completely assess the overall effects of hand hygiene use on the microbiome. Our report represents an important first step toward the goal of a standardized hand hygiene method incorporating culture-independent methods.

## MATERIALS AND METHODS

### Participants.

**FIG 4:**
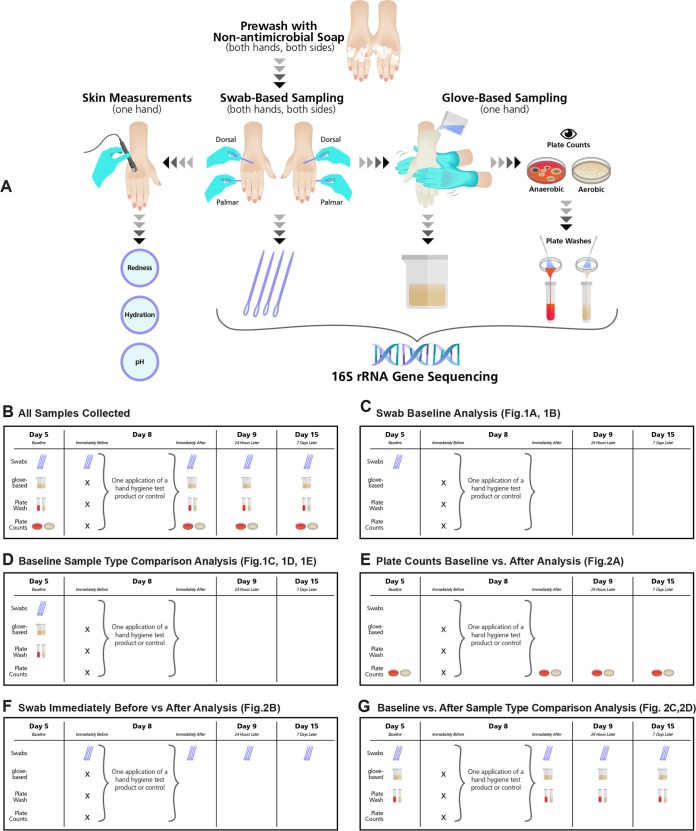
Study methodology. (A) Hand samples and measurements taken are visualized. First, hands were prewashed to remove transient microorganisms. Next, swab samples were taken on both sides of both hands, and then skin measurements were taken from one hand on the dorsal side, while the other hand was sampled using the glove-based method. A portion of each glove-based sample was directly sequenced, and another portion was plated aerobically and anaerobically. Colonies were counted, the growth from the plates was collected, and the plate washes were sequenced. (B) On study days 5, 9, and 15, all sample types were collected. On day 8, only swab samples were collected immediately before hygiene treatment, and all sample types were collected after treatment. Illustrations of which samples were used in the various comparative analyses among sample types collected on different days of the study are shown in panels C to G.

The protocol was approved by the Gallatin Institutional Review Board (Bozeman, MT) prior to subject enrollment. Subjects provided informed consent and agreed to follow the requirements of the study (see Fig. 4A for study methodology). Data were obtained from 50 participants who completed the entire study. Participants were Caucasian females (18 to 29 years old) in the area of Bozeman, MT. Standard previously published inclusion/exclusion criteria were followed (ASTM E1115; http://www.astm.org), with the addition of the following requirements: no nail treatments (nail polish, artificial nails, or use of nail polish remover), no immune system-compromising conditions, and no use of drugs known to affect the immune system or systemic antibiotics in the previous 90 days. Test subjects were randomly assigned to have one hand used for glove-based sampling and the other hand used for skin measurements throughout the course of the study. Swab-based samples were taken each time from the palmar and dorsal sides of both hands.

### Test materials.

Four test materials were evaluated in the study. The leading ABHS (PURELL Advanced Instant Hand Sanitizer) and nonantimicrobial hand wash (Softsoap Crisp Cucumber and Melon Hand Soap) in the consumer market in the United States (based on IRI data for a period ending 5 October 2014) were chosen for the test. The ABHS contained 70% ethanol as the active ingredient. Two controls, 70% ethanol–deionized water and a tap water rinse, also were evaluated. Products were used one time on day 8. Subjects were randomly assigned to use one of the four test materials.

### Study controls for factors outside product use.

Subjects participated in the study in November 2014 for 15 days, which included five visits to the testing laboratory (BioScience Laboratories, Inc., Bozeman, MT) on days 1, 5, 8, 9, and 15. In order to reduce the effects of the subjects’ personal product use, this study included a controlled washout phase from day 1 through day 5. On day 1, subjects were provided a personal product kit containing Pantene 2-in-1 Shampoo with Conditioner for hair washing, Suave (nonantimicrobial) Body Wash for bathing, and Softsoap Crisp Cucumber and Melon Hand Soap for hand washing. Throughout the study, subjects were not allowed to use their own personal products and were required to use only the products provided in their hygiene kit. They also were instructed to avoid hand contact with all antimicrobials and were not permitted to use lotion. Subjects were provided gloves to wear when circumstances necessitated use of an antimicrobial product or a product not provided in the personal product kit.

### Study timeline.

On day 1, subjects began following the study participation requirements, including the avoidance of all antimicrobials on the hands. Hands were not sampled on day 1. On day 5, hand measurements were performed to assess baseline conditions. On day 8, hands were first swabbed to obtain a sample immediately before product use, and then a hygiene test product or control was applied and hand measures were performed immediately after test product use. On days 9 and 15, hand measures were obtained to evaluate effects 24 h and 7 days after test product use, respectively. Figure 4B illustrates the types of samples collected on each of the days during which hands were sampled.

### Sample collection and hand measurements on study days 5, 9, and 15.

On all test days when hands were measured, subjects were instructed to not wash hands, shower, or bathe during the 2-h period prior to the hand sampling appointments. Upon arrival at the laboratory on each sampling day (days 5, 8, 9, and 15), subjects’ hands were washed with a nonantimicrobial hand wash (Softsoap Crisp Cucumber and Melon Hand Soap) to remove the transient bacteria and patted dry with sterile paper towels. Subjects then waited at least 15 min without touching any surfaces prior to sampling.

On days 5, 9, and 15, the palmar and dorsal areas of both hands were swabbed, followed by an additional 15-min waiting period without touching any surfaces. For the swabbing procedure, a sterile double swab (BD BBL CultureSwab; catalog number 220135) was rubbed for 60 s over the dorsal side of the hand, flipping the swab at 30 s and avoiding the fingernails. This was repeated with a new double swab on the palmar side of the hand. After a 15-min waiting period postswabbing, skin measurements were taken on the non-glove-based sampled hand to assess skin color, moisture content, and pH. For all skin measurements, three readings were taken from the dorsal side of the hand and the measurements were averaged, with pH always assessed last. Skin color was assessed using a Konica Minolta CR-400 Chroma Meter. Measurement of skin moisture content was performed using an MPA 6 system with a Courage-Khazaka CM 825 Corneometer probe. pH was measured using an MPA 6 system with a Courage-Khazaka PH 905 skin pH meter probe on the skin, which was first hydrated by adding a few drops of distilled water to the skin before each measurement. Immediately following skin measurements, glove-based sampling of the other hand (randomized; one hand was used for skin measures and the other hand for glove-based sampling) was conducted as described in ASTM E1115 (http://www.astm.org), with 50 ml of a modified sterile hand sampling solution (0.5% K_2_HPO_4_, 0.4% Na_2_HPO_4_, 0.1% Tween 80, 0.03% l-cysteine HCl prepared in deionized water, pH 6.8 ± 0.1). The modified solution was used instead of the solution suggested in the ASTM E1115 and E1174 protocols because the Triton X-100 in the standard formulation is known to prevent the growth of some normal skin bacteria (43), and pilot evaluations (data not shown) demonstrated higher bacterial recovery counts from hands using the modified formulation than using the standard hand sampling solution.

### Hand hygiene treatment, sample collection, and hand measurements on study day 8.

On day 8, each participant received a treatment consisting of one dose of a hygiene test product or control. After completing the nonantimicrobial hand wash and the 15-min waiting period upon entering the testing facility, both of the subjects’ hands were swabbed as previously described. After another 15-min waiting period, non-glove-based skin measurements of the sampled hand were taken as previously described. The test article was applied once to the subjects’ hands. The ethanol control and ABHS were applied in 1.5-ml volumes and rubbed in until the hand was dry. The hand wash and the water control were applied in 1.5-ml volumes to wet hands, washing was performed for 15 s, rinsing was performed for 10 s, and the hands were patted dry with sterile paper towels. Immediately after application of the test product, both of the hands of each subject were swabbed. The other (nontreated) hand of each subject was then sampled with the glove-based method.

### Sample handling and storage.

Within minutes after collection, the swabs were transferred to sterile cryovials and put on dry ice. Within approximately 60 min, the samples were placed at −70°C. The hand sampling solution used in the glove-based method was transferred to a 50-ml centrifuge tube and adjusted to 40 ml total. A 10-ml aliquot was removed for culture plating, and the remaining 30 ml was centrifuged at 4,500 × *g* for 20 min at 4°C to concentrate the biomass in the sample. The pellet was resuspended in 1.6 ml of diethyl pyrocarbonate (DEPC)-treated water and divided into four equal aliquots, each of which was stored in a 1.8-ml cryovial at −70°C. Samples were shipped overnight on dry ice for DNA analysis.

### Sample results used in the different comparative analyses.

The four different swab samples taken at baseline (dorsal dominant, dorsal nondominant, palmar dominant, and palmar nondominant) were compared within and between participants in one analysis (Fig. 4C). In another comparison, sequencing results from swab-based samples, glove-based samples, and plate washes taken at day 5 (baseline) were compared (Fig. 4D). Figure 4E shows the plate counts used to perform the baseline versus posttreatment analyses. Data from the samples used for analysis of swab-based results taken immediately before versus after the hygiene intervention are shown in Fig. 4F. In another analysis, baseline glove-based samples, swab-based samples, and plate wash samples were compared to the same sample types taken immediately, 24 h and 7 days after hygiene treatment (Fig. 4G).

### Plate culture analysis.

The hand sampling solution used for the glove-based method was used for plating of duplicate 50-μl spiral plates on tryptic soy agar for recovery of aerobic viable bacteria and on tryptic soy agar with 5% sheep’s blood for recovery of anaerobic viable bacteria. Aerobic plates were incubated at 35°C for 3 days, and anaerobic plates were incubated at 35°C for 5 days in anaerobic jars. All plates were counted using a computerized counting system. Log_10_ CFU reductions were calculated by subtraction of the number of viable microorganisms recovered following product usage from the number of viable microorganisms recovered at baseline. In addition, a postculture plate wash was obtained by rinsing agar plates with 2 ml of the hand sampling solution to collect the viable microorganisms from the surface of the agar.

### 16S rRNA gene sequencing and analysis.

Sequences from swab-based samples were collected in two Illumina MiSeq sequencing runs, and glove-based samples were collected in one Illumina MiSeq sequencing run using 2 × 150-bp paired-end sequencing kits and techniques similar to those previously described for the V4 region by Fierer et al. (23). Raw sequences were demultiplexed using a Python script (https://github.com/leffj/helper-code-for-uparse), the resulting sequences were clustered, and OTU abundances were calculated on a per-sample basis using the UPARSE pipeline. The sequence processing consisted of building a *de novo* sequence database from the demultiplexed paired-end sequences, which were merged and quality filtered using a maximum error rate of 0.5 bp per sequence. Singleton sequences were removed prior to building the database, and the remaining sequences were clustered using a >97% sequence similarity cutoff to form OTUs. Representative sequences of these OTUs (the *de novo* database) were provided taxonomic identifications using the RDP classifier trained on the Greengenes database (version 13_8). To obtain sequence counts for each OTU per sample, merged unfiltered reads were mapped to the *de novo* database. OTUs identified as mitochondria or chloroplasts were removed prior to downstream analyses. Since blanks included among the samples during microbial community analysis were consistently found to contain members of the genus *Alicyclobacillus*, OTUs classified as *Alicyclobacillus* were removed also prior to downstream analyses. All samples were rarefied to 1,000 sequences per sample.

### Statistical analyses.

Differences in community composition across samples were represented by Bray-Curtis dissimilarities calculated from square root-transformed OTU abundances. Effects of subject, hand side (palmar versus dorsal), and dominant versus nondominant hand on bacterial diversity were assessed using analysis of variance (ANOVA), and effects on community composition were assessed using permutational multivariate analysis of variance (PERMANOVA), including all explanatory variables in the model. Differences in diversity and differences in community composition across sample types (swab-based sequences, glove-based sequences, and sequences of plate washes from aerobic and anaerobic culture growth) were assessed using ANOVA and PERMANOVA, respectively. Effects of hand hygiene treatment on skin bacterial community composition were assessed by calculating dissimilarities between pretreatment samples and posttreatment samples for the same individual and for the same hand (left versus right), as well as for the sides of the hand (palmar versus dorsal) for swab samples. ANOVA was used with Tukey’s honestly significant difference (HSD) tests with the predictor variables treatment, dominant/nondominant hand (for swab-based samples), and hand side (for swab-based samples) to determine whether there were significant differences across treatments at each time point.

Statistical analysis and data manipulation were conducted with scripts written in R (44) with the aid of the “mctoolsr” (https://github.com/leffj/mctoolsr), “vegan,” “dplyr,” “reshape2,” and “ggplot2” packages. Values representing the significance of differences in plate counts and log_10_ reductions were determined by one-way ANOVA for α = 0.05, using the Bonferroni *post hoc* multiple-comparison test (GraphPad Prism Software, Inc.).

Relationships between skin metrics and bacterial community composition were assessed by matching the swab-based sample collected from the dorsal side of the same hand in which the skin metrics were observed and using Mantel tests with Spearman correlations. Relationships between levels of culturable bacteria and bacterial community composition also used Mantel tests as described above. Correlations among the skin measures, plate counts, and log_10_ reductions for the time point immediately after hygiene treatment were identified using linear regression, which reports significance as well as the direction of correlation (positive or negative) (GraphPad Prism Software, Inc.).

### Data availability.

The raw DNA sequence data are publicly available on figshare: https://doi.org/10.6084/m9.figshare.c.3709684.v1.
